# Editorial Note

**Published:** 1913-12

**Authors:** 


### Editorial Note.

**The Training of Nurses.**

FROM the New York State Education Department a *Report on Higher Education* for the year ending July, 1912, has just been published. The scientific investigation of methods and results in teaching which has been carried out for some years past in the United States of America has earned the admiration and gratitude of all the world. The present report deals with many branches of education, but the section which we desire to single out for comment and commendation is that upon the education of hospital nurses. Since the passing of the Nurse Practice Act in 1903, providing for the registration of nurses, gradual progress has been made towards the standardisation of nurses' training in hospitals, and by slow collection of experience the Education Authorities (the "Regents") of New York State have endeavoured to place the various training schools upon a sound professional basis.

A most important measure was adopted in 1906, namely the requirement of proof of at least a course of one year in a secondary school. Acting with cautious wisdom, this evidence of preliminary education was interpreted at first somewhat loosely, but the conclusion arrived at is that "the dearth of pupils appears to be greater in the schools of poor standards of admission, etc., than in those of higher requirements." Complaint was made that "the restriction of the admission of probationers is working a hardship on hospitals which are conducting properly equipped and ethically administered training schools." The reply has a fine directness about it: "No school can be considered as ethically conducted which would break down standards which are not excessive for the purpose of securing a sufficient number of probationers to maintain an unpaid nursing service." The "Regents" realise this, and in the face of opposition maintain that the training of nurses as a branch of education requires to be considered quite apart from the immediate supply of attendants upon the sick in any particular hospital. This to English ideas is an almost new view point. The report expresses great concern as regards *the overstrain of nurses.* It is urged that the hours on duty are too long, averaging sixty-two hours weekly on day duty, and eighty hours on night duty; that the proportion of time allotted to theoretical instruction is insufficient, and that this instruction takes place either during the so-called hours of recreation or during the evening; and that except in a few schools that have established preliminary courses no time is allowed for study. The main part of the criticisms is applicable to our British hospitals. So too is the following indictment: "The age in many of the schools has fallen in the past few years from 23 to 19, 18 and even 17. That pupils so immature should be placed after a few months in the hospital, frequently less than three, in sole charge of from ten to sixty patients, and without relief for eleven to twelve hours nightly, is little short of cruelty. Such long hours and such responsibility produce not only physical weakness but a dulness of the perception and sensibilities, with a resultant deterioration in the quality of the service rendered, and are therefore as detrimental to the patients' welfare as to the pupils'."

These are strong words of the New York "Regents," but they are none too strong. With a few praiseworthy exceptions, the condemnation falls equally upon our English hospitals, and it behoves the authorities of our more enlightened training schools, as well as the medical profession, to exert their united endeavours to bring about a long overdue reform.

The enthusiasm of the Americans for sound education is no new upstart. We shall be following no ill leaders if we adopt their reforming zeal, and clothe ourselves with a corner of the mantle inherited from that General Court of Massachusetts which in 1647 established schools in every township, "to the end that learning may not be buried in the graves of the forefathers."

